# Contribution of Rare Copy Number Variants to Isolated Human Malformations

**DOI:** 10.1371/journal.pone.0045530

**Published:** 2012-10-03

**Authors:** Clara Serra-Juhé, Benjamín Rodríguez-Santiago, Ivon Cuscó, Teresa Vendrell, Núria Camats, Núria Torán, Luis A. Pérez-Jurado

**Affiliations:** 1 Unitat de Genètica, Universitat Pompeu Fabra, Barcelona, Spain; 2 Centro de Investigación Biomédica en Red de Enfermedades Raras (CIBERER), Barcelona, Spain; 3 Quantitative Genomic Medicine Laboratories (qGenomics), Barcelona, Spain; 4 Programa de Medicina Molecular i Genètica, Hospital Universitari Vall d'Hebron, Barcelona, Spain; 5 Servei d'Anatomia Patològica, Hospital Universitari Vall d'Hebron, Barcelona, Spain; Instituto de Ciencia de Materiales de Madrid - Instituto de Biomedicina de Valencia, Spain

## Abstract

**Background:**

Congenital malformations are present in approximately 2–3% of liveborn babies and 20% of stillborn fetuses. The mechanisms underlying the majority of sporadic and isolated congenital malformations are poorly understood, although it is hypothesized that the accumulation of rare genetic, genomic and epigenetic variants converge to deregulate developmental networks.

**Methodology/Principal Findings:**

We selected samples from 95 fetuses with congenital malformations not ascribed to a specific syndrome (68 with isolated malformations, 27 with multiple malformations). Karyotyping and Multiplex Ligation-dependent Probe Amplification (MLPA) discarded recurrent genomic and cytogenetic rearrangements. DNA extracted from the affected tissue (46%) or from lung or liver (54%) was analyzed by molecular karyotyping. Validations and inheritance were obtained by MLPA. We identified 22 rare copy number variants (CNV) [>100 kb, either absent (n = 7) or very uncommon (n = 15, <1/2,000) in the control population] in 20/95 fetuses with congenital malformations (21%), including 11 deletions and 11 duplications. One of the 9 tested rearrangements was *de novo* while the remaining were inherited from a healthy parent. The highest frequency was observed in fetuses with heart hypoplasia (8/17, 62.5%), with two events previously related with the phenotype. Double events hitting candidate genes were detected in two samples with brain malformations. Globally, the burden of deletions was significantly higher in fetuses with malformations compared to controls.

**Conclusions/Significance:**

Our data reveal a significant contribution of rare deletion-type CNV, mostly inherited but also *de novo*, to human congenital malformations, especially heart hypoplasia, and reinforce the hypothesis of a multifactorial etiology in most cases.

## Introduction

A potentially lethal or disabling major malformation occurs in 2–3% of liveborn infants and 20% of stillborn fetuses [1]. Congenital malformations have become the main cause of infant mortality during the first years of life [2] and are associated with long term morbidity [3], [4]. In particular, congenital heart defects (CHD) represent a high percentage of clinically significant birth defects. The incidence of CHD is approximately 8 per 1,000 livebirths making CHD the most common malformation [5], [6].

Congenital malformations often occur in the setting of multiple congenital anomalies, including dysmorphic facial features, developmental aberrations of different organs, or growth abnormalities [7], [8]. In these cases with a more complex syndrome, chromosomal aberrations are a frequent cause of disease, although point mutations in developmental or metabolic genes have also been described in specific syndromes [9], [10]. Standard karyotyping can detect numerical and structural anomalies larger than 5–10 Mb and other techniques, such as fluorescent in situ hybridization (FISH) [11] or MLPA [12]–[14], allow the identification of submicroscopic chromosomal imbalances. In the last decade, the development of molecular karyotyping by array comparative genomic hybridization (aCGH) or single-nucleotide-polymorphism (SNP) microarrays, globally termed chromosomal microarray analysis (CMA), has allowed the detection of as much as 15–24% of causative segmental aneusomies in patients with multiple congenital anomalies and/or intellectual disability [15], [16]. Retrospective studies in fetuses with multiple malformations have obtained a detection rate of causative chromosomal imbalances from 8 to 15% by using CMA [17]–[19], and the clinical utility of a targeted CMA has been demonstrated in standard invasive prenatal diagnosis [20], [21]. CHD are among the malformations in which genomic rearrangements have been shown to play a major role. For instance, microdeletions at 22q11.2 [22], [23] and microduplications at 1q21.1 [24], [25] are a common cause of conotruncal heart defects.

In an important proportion of cases, only one malformation is detected without the presentation of other minor or major defects. Although some isolated congenital malformations can be caused by environmental risk factors, such as maternal diseases or exposure to teratogenic agents during pregnancy [4], there is strong evidence that genetics plays a major role, as epidemiological studies have shown an increased risk of this type of anomalies in siblings and offspring of individuals with sporadic congenital malformations, as well as increased paternal age and high concordance in monozygotic twins [26]–[28]. A small percentage can be attributed to point mutations in development related genes [29], [30], although this type of genetic alterations have been insufficiently tested until recently. Submicroscopic deletions and duplications may play a significant role in the etiology of this condition, either as direct cause or as possible genetic risk factor for isolated congenital anomaly [31]. Nevertheless, the mechanisms underlying the majority of non-chromosomal or sporadic congenital malformations are poorly understood.

Finding the cause of congenital malformations is necessary to better understand the pathophysiological basis of these developmental anomalies and define disease risks, both critical elements to ensure proper genetic counseling and disease prevention. Genetic counseling has become more relevant in this area considering not only the recurrence risk of healthy parents after having an index case, but also that more individuals with congenital malformations are living into adulthood due to advances in medical and surgical care and may have the opportunity to reproduce [7].

We have searched for cryptic genomic rearrangements in fetuses with isolated congenital malformations and fetuses with more than one congenital anomaly. Our data illustrate a significant contribution of rare deletion-type CNV, mostly inherited but also *de novo*, to human congenital malformations. These genomic rearrangements could represent the single genetic etiology of the disease, perhaps as part of a more complex syndrome without other recognizable manifestations at this stage of development, or genetic susceptibility factors contributing to the mutational load in multifactorial disorders.

## Methods

### Ethics Statement

All studies were performed as part of an expanded diagnostic protocol approved by the Medical Ethical Committee of the Vall d'Hebron Hospital, after receiving written informed consent from the family.

### Samples/Patients

Fetuses were selected from medically terminated pregnancies between 17 and 22 weeks of gestation owing to one or more malformations with bad prognosis detected during pregnancy. Samples were collected from frozen tissues stored in the Tissue Bank of Vall d'Hebron Hospital. A complete fetopathological examination had been performed and the samples were classified in two different groups: 1) 68 samples with an isolated congenital malformation, including 33 with isolated CHD, 26 with isolated central nervous system (CNS) malformation and 9 with isolated renal malformation; 2) 27 fetuses with more than a unique malformation. Prenatal GTG banding chromosome analysis was normal for all 95 fetuses. An overview of the clinical features of the fetuses included in the study is summarized in table 1 (detailed in Tables S1, S2, S3, S4).

**Table 1:** Overview of malformations in the 95 analyzed fetuses.

MALFORMATION	SAMPLES
**Congenital heart disease**	
Conotruncal defect	13
Heart hypoplasia	17
Other	3
**Central nervous system malformation**	
Neural tube defect	16
Holoprosencephaly	3
Hydrocephalus	3
Ventriculomegaly	3
Agenesis of the corpus callosum	1
**Renal malformations**	
Agenesis	5
Dysplasia	3
Nephronophthisis	1
**Multiple malformations**	27

Parental blood samples were collected in cases in which an alteration was identified.

### DNA extraction from tissue and blood samples

In fetuses with an isolated congenital malformation, the affected tissue (heart, brain or kidney) was obtained when available (n = 44); liver or lung tissue was used for the remaining samples with insufficient target tissue (n = 24). For fetuses with multiple congenital anomalies (n = 27), liver or lung tissue was used. Parental DNA was isolated from total blood. DNA was extracted using the Gentra Puregene Blood kit (Qiagen) according to manufacturer's instructions.

### Multiplex Ligation-dependent Probe Amplification (MLPA)

Genomic rearrangements in subtelomeric regions (P036 and/or P070, MRC Holland) as well as recurrent microdeletion or microduplication syndromes (custom made, Table S5) were also discarded prior to selection by using two MLPA panels.

An MLPA assay was also designed to validate the genomic alterations detected by CMA and to study the inheritance in those cases with available parental samples. A total of 100 ng of genomic DNA from each sample was subject to MLPA using specific synthetic probes [Table S6] designed to target the specific CNV detected by different types of array. All MLPA reactions were analyzed on an ABI PRISM 3100 Genetic analyzer according to manufacturers' instructions. Each MLPA signal was normalized and compared to the corresponding peak height obtained in control samples [32], [33].

### Molecular karyotyping by CMA

The entire cohort was studied by using BAC (Bacterial Artificial Chromosome) aCGH. DNA samples (1 µg) were labeled by random priming with Cy3-dCTP and Cy5-dCTP and hybridized against a reference pool of the same gender. Samples were hybridized onto a BAC aCGH containing 5,600 clones with a backbone mean coverage of ∼1 Mb and increased density in hotspot regions for genomic rearrangements (subtelomeres, pericentromeres and regions flanked by segmental duplications). Analyses of BAC-aCGH data were performed as previously described [32].

A total of 25 samples were also studied by using an oligonucleotide Agilent H244K aCGH. Samples were processed and hybridized according to manufacturer's recommendations (Agilent Protocol v6.0, ref. G4410-90010). This technique allowed us to validate and better map the breakpoints of the alterations detected by BAC aCGH, as well as to increase the resolution of the study in samples in which no alteration had been detected using BAC aCGH. Only CNVs with genes, longer than 100 kb and with a frequency in control samples lower than 1/2,000 were considered. The frequency of each CNV in the control population was determined using 1 M Illumina SNP array data from a control database of 8,329 samples already reported [34], along with data from 1,991 Spanish adult samples from the Spanish Bladder Cancer/EPICURO study including 1034 patients with urothelial cell carcinoma of the bladder and 957 hospital-based generally healthy controls with a mean age of 63.7 years [35].

DNA from 70 samples was studied by using the 370K Illumina SNP array. This technique permitted us to increase the resolution in samples in which no alteration had been identified using BAC aCGH. Moreover, using SNP array uniparental disomy and regions with high level of homozigosity were studied. Copy number changes were identified using the PennCNV software with stringent filtering, as previously described [35]. Only CNVs with genes, longer than 100 kb and with a frequency in control samples lower than 1/2,000 were considered. A search for possible mosaic copy number and copy neutral changes was also performed using the MAD algorithm [36].

### Genetic counseling

Genetic counseling was offered to all couples when an alteration was identified in order to explain the findings and the need for further testing including parental samples. After the study of the parents' samples, follow-up counseling was provided along with a written report explaining the alteration, the putative relation with the phenotype and the implications to the family.

### Bioinformatic and statistical analyses

The frequency of each CNV in the population was determined using 1 M Illumina SNP array data from a control database of 8,329 samples already reported [34], along with data from 1,991 Spanish adult samples studied in our laboratory with the same arrays [35].

In addition, already available data from a randomly selected cohort of 168 generally healthy Spanish adult control individuals (Spanish Bladder Cancer/EPICURO study genotyped with Illumina 1 M SNP array [35]) was used in order to compare the different frequencies of rare rearrangements between controls and fetuses with congenital malformations (global CNV burden and CNV combinations). In order to avoid or minimize a possible bias due to the different detection yield of the array platforms used, we only considered alterations larger than 100 kb that should be detected with any of the platform arrays. For the comparative analyses, only CNVs with genes, a minimum length of 100 kb and a frequency in control samples lower than 1/2,000 were considered. Alterations totally overlapping with segmental duplications were also excluded to minimize biases due to the different probe coverage among microarray platforms.

### Gene content and enrichment analyses

The gene content (genes included or disrupted) of the rare CNVs identified in the cohort of fetuses was analyzed using a computational resource, Consensus Path DB [37], to obtain an overview of the pathways that could be altered. Pathways were considered overrepresented when their p-value was above 0.05.

## Results

**Figure 1:**
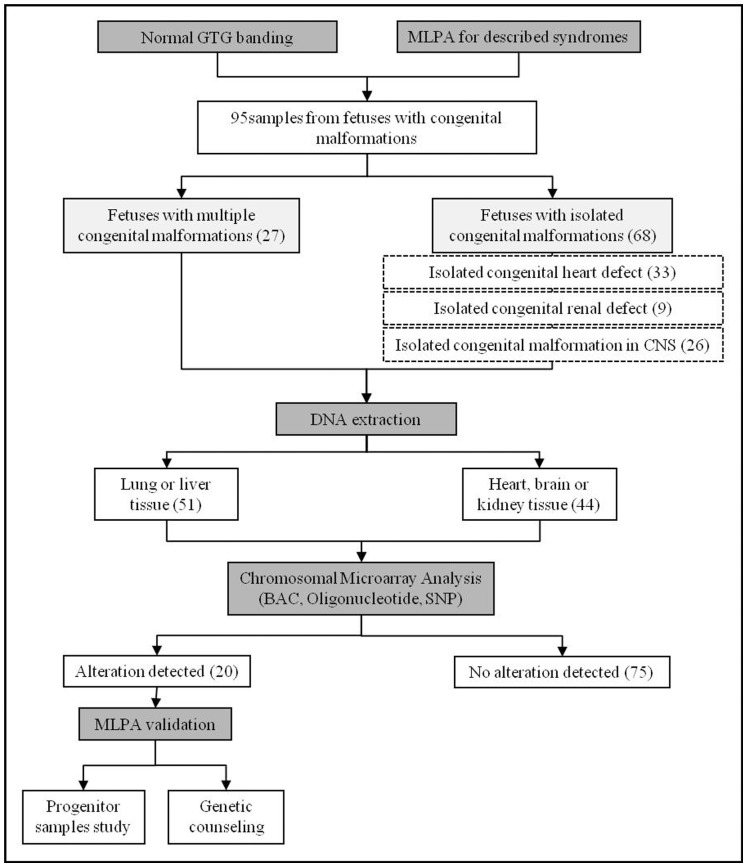
Strategy followed to study samples of fetuses with congenital malformations. MLPA: multiplex ligation-dependent probe amplification; CNS: central nervous system; BAC: bacterial artificial chromosome; SNP: single nucleotide polymorphism.

Prenatal GTG banding chromosome analysis was normal for all 95 fetuses. Known microdeletion/microduplication syndromes and subtelomeric genomic rearrangements were also discarded by MLPA in all cases. All samples were first studied using BAC aCGH and then by oligonucleotide or SNP array (Fig. 1).

Globally, CMA detected 22 CNVs fulfilling the established criteria (>100 kb, gene containing and present in <1/2000 controls) in 20 samples (21.05%), 11 deletions and 11 duplications (100.6–2,324 kb in length), with 2 samples harboring two rearrangements. MLPA probes were designed to define the inherited or *de novo* nature of the CNVs in all 9 cases from whom parental samples were available. In 8 cases the alterations were inherited, while the rearrangement was *de novo* in a single case. The detected alterations are listed in table 2, including information about the genomic coordinates, size, microarrays used for detection and validation, inheritance and genes included in the region. Among the 22 alterations identified, 7 (4 duplications and 3 deletions) have never been found in the 10,320 adults used as controls. Two aberrations, both of them identified in fetuses with CHD, overlap with previously reported alterations associated with developmental anomalies and are likely the underlying genetic cause [38]–[40]: 1) A 363 kb *de novo* deletion in 16q24.1, encompassing five genes (*FOXF1, FOXC2, MTHFSD, FLJ30679* and *FOXL1)*, was detected in a fetus with left heart hypoplasia (case 2); 2) the recurrent 2.2 Mb 15q13.3 deletion was identified in a fetus with right heart hypoplasia as well as in the healthy mother (case 1). The remaining 20 rearrangements have not been described in patients with disease.

**Table 2:** Summary of copy number variations detected in 95 fetuses with congenital malformations.

Case #	Malformation	Gain/Loss	Region	Length (kb)	Start	End	Array used	Inheritance	Genes in the region	Control frequency (10,320)
**6**	CHD	Gain	5q35.2	297.3	175798945	176096236	SNP	-	*ARL10, CLTB, EIF4E1B, FAF2, GPRIN1, HIGD2A, NOP16, PCDH24, RNF44, SNCB, TSPAN17*	0
**2**	CHD	Loss	16q24.1	363.5	86382121	86745576	SNP	*De novo*	*FOXF1, FOXC2, MTHFSD, FLJ30679, FOXL1*	0
**4**	CHD	Loss	16q23.3	120.3	83869776	83990089	SNP	Paternal	*MLYCD, OSGIN1*	1
**23**	CHD	Loss	6p25.1	139.4	5249765	5389206	Oligonucleotide	-	*LYRM4, FARS2*	1
**24**	CHD	Gain	2p25.3	108.5	3579585	3688127	SNP	-	*COLEC11, RNASEH1, RPS7*	1
**29**	CHD	Gain	3p26.3	246.5	2869944	3116438	SNP	Paternal	*CNTN4, IL5RA*	1
**1**	CHD	Loss	15q13.3	2207	30755144	32962148	BAC and Oligo	Maternal	*FAN1, MTMR10, TRPM1, LOC283710, KLF13, OTUD7A, CHRNA7*	1
**3**	CHD	Loss	13q21.2	288.1	60410392	60698463	BAC and SNP	Maternal	*DIAPH3*	2
**15**	CHD	Gain	10q26.3	181.4	134572478	134753880	Oligonucleotide	-	*INPP5A, NKX6-2, TTC40*	2
**13**	CHD	Gain	9p21.1	369.2	28659143	29028380	SNP	-	*LINGO2*	4
**48**	CNS	Loss	17p12	1383.3	14090300	15473646	Oligonuclotide	-	*COX10, CDRT15, HS3ST3B1, PMP22, TEKT3, CDRT4, FAM18B2*	0
**57**	CNS	Loss	6p22.2	112.9	24401654	24514569	SNP	Maternal	*ALDH5A1, GPLD1, MRS2*	0
		Loss	12p12.3	311.6	18337494	18649057	SNP	Paternal	*PIK3C2G*	4
**50**	CNS	Gain	1p33	139.2	46814268	46953453	Oligonuclotide	-	*FAAH, DMBX1, KNCN*	0
		Gain	10q11.22	197.3	46951237	47148490	Oligonuclotide	-	*SYT15, GPRIN2, PPYR1*	4
**59**	CNS	Gain	5p13.2	150.3	37411054	37561355	BAC and Oligo	-	*WDR70*	4
**53**	CNS	Loss	18q22.1	2324.0	63733025	66057032	SNP	Maternal	*CDH19, DSEL, LOC643542*	4
**65**	Renal	Loss	4q12	883	52798624	53681594	SNP	Maternal	*DANCR, LRRC66, SGCB, SNORA26, SPATA18, USP46*	1
**72**	Multiple	Gain	10p14	128.4	11815455	11943885	SNP	-	*C10orf47, LOC219731*	0
**82**	Multiple	Gain	5q35.3	752.9	179833485	180586413	SNP	-	*BTNL3, BTNL8, BTNL9, CNOT6, FLT4, LOC729678, MGAT1, OR2V2, OR2Y1, SCGB3A1, ZFP62*	0
**85**	Multiple	Loss	7p14.1	130.1	40264889	40394987	SNP	-	*C7orf10*	1
**84**	Multiple	Gain	1p34.1	100.6	46252717	46353332	SNP	-	*MAST2*	5

Control frequency refers to the frequency of the same type of rearrangement found in the fetus, deletion or duplication. Hg19 assembly. CHD: congenital heart defect; CNS: central nervous system; SNP: single nucleotide polymorphism; BAC: bacterial artificial chromosome.

Although not included in the listed 22 aberrations because its reported frequency in controls is 0.14% (>1/1,000), we also detected the recurrent 1.6 Mb 16p13.11 duplication in two samples, one case of CNS malformation (neural tube defect and Arnold-Chiari malformation) and another with multiple malformations (anal imperforation, right heart hypoplasia and esophagus atresia). The reciprocal deletion of this region has been clearly associated with increased risk for congenital malformations and developmental difficulties but published data for the duplication are not clearly conclusive [41].

In order to define whether the global burden of rare CNVs in the fetuses with congenital malformations was or not significantly increased, we compared it with a cohort of 168 control subjects analyzed with the Illumina 1 M SNP array. For consistency, only CNVs larger than 100 kb, containing genes, not totally overlapping with segmental duplications, and found at a frequency <1/2,000 were considered (listed in Table S7). Rare CNVs fulfilling criteria were identified in 17.86% of the control samples including 2 samples with 2 alterations. These rare CNVs in controls were predominantly duplications (78.12% vs 21.88% deletions). Thus, the global CNV burden in malformed fetuses was only slightly increased with respect to that in normal controls (21.05% vs 17.86%).

The proportion of samples with rearrangements was different between the different groups of malformations, being higher in fetuses with CHD (10/33 samples, 30.30%) and even higher if only heart hypoplasia was considered (8/17, 47.06%). The difference in aberration frequency between groups was statistically significant comparing fetuses with heart hypoplasia and controls (p = 0.009). The difference in the frequency of deletion-type CNV between cases and controls was also statistically significant (50% vs 21.88%, p = 0.03) and more evident comparing only fetuses with heart hypoplasia and controls (p = 0.001). These differences were due to the increased number of deletions, but not duplications, in cases with congenital malformations (Table 3). The frequency of individuals with more than one CNV hit fulfilling the established criteria was not different between cases and controls, around 2% (Table 3).

**Table 3:** Comparisons of rare copy number changes >100 kb detected in the fetuses with congenital malformations and controls.

GROUP	ALTERATIONS	DELETIONS	DUPLICATIONS	DOUBLE HIT	SAMPLES
**Controls (168)**	32	7 (4.2%/21.88%)	25 (14.9%/78.12%)	2 (1.19%)	30 (17.86%)
**Fetuses (95)**	22	11 (11.6%/50%)	11 (11.6%/50%)	2 (2.11%)	20 (21.05%)
**CHD (33)**	10	5 (15.2%/50%)	5 (15.2%/50%)	0 (0%)	10 (30.30%)
*Heart hypoplasia (17)	8	5 (29.4%/62.5%)	3 (17.6%/37.5%)	0 (0%)	8 (47.06%)
**CNS malformations (26)**	7	4 (15.4%/57.14%)	3 (11.5%/42.86%)	2 (7.69%)	5 (19.23%)
**Renal malformations (9)**	1	1 (11.1%/100%)	0 (0%/0%)	0 (0%)	1 (11.11%)
**Multiple malformations (27)**	4	1 (3.7%/25%)	3 (11.1%/75%)	0 (0%)	4 (14.81%)

In brackets the proportion of samples with the CNV and the proportion of the specific type of rearrangement. *A subcategory of CHD only considering heart hypoplasia has been added to the table due to remark the different frequency of CNVs with respect to the other CHD. CHD: congenital heart defect; CNS: central nervous system.

**Figure 2:**
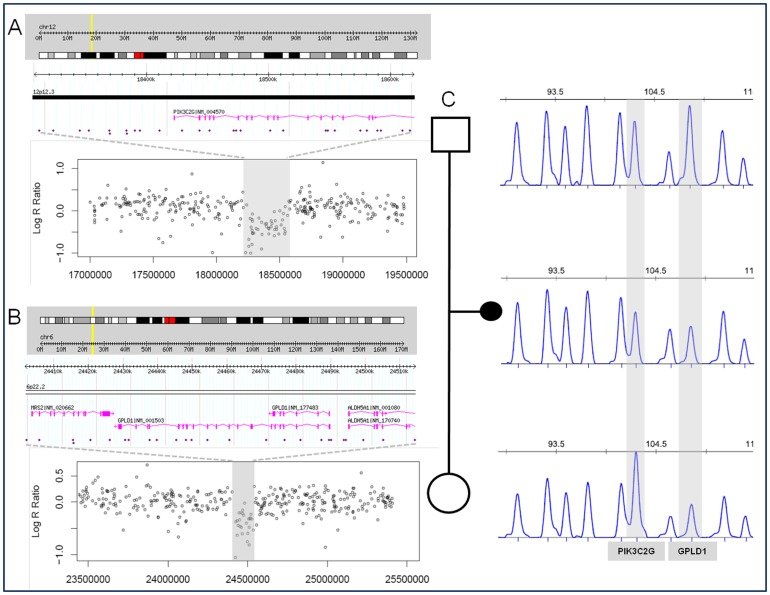
Detection, validation and inheritance of the two chromosomal deletions in case 57. A and B: Ideogram showing the location of the rearrangement and the corresponding regional plot of the Log R Ratio values of the SNP array (deleted and flanking regions). C: MLPA pattern in the familial trio showing the inheritance of both deletions. Hg19 assembly. MLPA: multiplex ligation-dependent probe amplification; SNP: single nucleotide polymorphism.

Regarding the overrepresentation analysis, phosphatidylinositol phosphate metabolism was the only pathway significantly overrepresented in cases with respect to controls. Three genes directly involved in this pathway, *PIK3C2G*, *GPLD1* and *INPP5A*, are included in the CNVs identified in two fetuses. Interestingly, two of these genes are located in two deletions found in the same sample, a fetus with holoprosencephaly. One deletion encompassing three genes, *ALDH5A1*, *GPLD1* and *MRS2*, was inherited from the mother, while the other one including only one gene, *PIK3C2G*, was inherited from the father (Fig. 2). An additional sample with two events was a fetus with hydrocephalus found to have two duplication CNVs, on chromosome bands 1p33 (including the genes *FAAH*, *DMBX1* and *KNCN*) and 10q11.22 (containing the genes *SYT15*, *GPRIN2* and *PPYR1*), but parental samples were not available in this case.

No large stretches of homozygosity suggestive of parental consanguinity or uniparental disomy (UPD) were identified in any sample (70/95 fetuses studied with SNP arrays). In addition, despite the use of DNA from the affected tissue in 46% of cases, no events of copy number or copy neutral changes suggestive of somatic mutations were detected.

## Discussion

Chromosomal aberrations have been reported as a frequent cause of congenital malformations, especially when they are associated with growth or developmental delay, malformations affecting a second organ or dysmorphic features [6], [16], [18], [42]. Many of the chromosomal unbalances associated with such syndromes are large and encompass multiple genes. A detection rate of 10% of chromosomal abnormalities, including one marker chromosome, one rearrangement of 9 Mb and another rearrangement of 13 Mb, has been reported studying by aCGH a population of 50 fetuses with at least three malformations or a severe brain anomaly [42]. A yield of 16.3%, considering known syndromes, was found in a cohort of 49 fetuses with birth defects [18]. The role of submicroscopic deletions and duplications in isolated congenital malformations has been documented for CHD with the identification of 18 putatively pathogenic CNVs (17.1%) in 105 samples from infants with isolated CHD [31], including recurrent rearrangements in 22q11.2 (responsible of DiGeorge syndrome), 17p11 (causative of Smith-Magenis syndrome) and 1q21.1, a large alteration of 14 Mb and an aberration with no genes.

In our series, chromosomal alterations detected by karyotyping and cryptic alterations in subtelomeric regions or known microdeletion/microduplication syndromes were previously excluded. Rare CNVs larger than 100 kb were detected in 21% of fetuses with prenatally detected malformations, with a yield of 30.3% in fetuses with CHD. The CNV burden was slightly but significantly higher in malformed fetuses compared with controls (21.05% vs 17.86%). Deletions were also more prevalent in cases than controls (50% vs 21.88%). As expected, large CNVs and mostly deletions are more likely to affect gene expression with relevant effect on developmental pathways. The difference in the detection rate in comparison with other studies might be explained by the different selection criteria and resolution of the array platforms used.

We detected abnormalities previously reported as causative of CHD in two cases. A 363 kb *de novo* deletion in 16q24.1 encompassing the *FOX* gene cluster was detected in a fetus with left heart hypoplasia. Overlapping deletions have been previously reported in patients with alveolar capillary dysplasia, misalignment of pulmonary veins and distinct malformations including congenital heart defect, specifically hypoplastic left heart [38]. Deletion of *FOXF1* is thought to be responsible for alveolar capillary dysplasia while *FOXC2* is related to the lymphoedema-distichiasis syndrome. Larger deletions, as in our case, may cause a more complex syndrome which includes CHD likely due to additive effects of haploinsufficiency for contiguous genes [38].

We also identified the recurrent 2.2 Mb 15q13.3 deletion in a fetus with right heart hypoplasia, inherited from the healthy mother. Interestingly, the brother of the mother also had a cardiac malformation on anamnesis but he rejected to be studied. Deletions and duplications at 15q13.3 have been related to different developmental anomalies, such as dysmorphic features, intellectual disability, seizures, schizophrenia, and in 17% of patients congenital heart defects [39]. Based on previous studies in animal models, *KLF13*, encoding the Kruppel-like factor 13, is the best candidate gene for the cardiac defects associated with the 15q13.3 deletion. *KLF13* knockdown in Xenopus embryos caused atrial septal defects and hypotrabeculation similar to those observed in humans or mice with hypomorphic *GATA4* alleles [40]. Rearrangements in this region show incomplete penetrance and variable expressivity, with various cases in which the deletion or duplication is inherited from a healthy progenitor, as in our case [39]. Given this incomplete penetrance of clinical manifestations and the relatively low proportion of patients affected by cardiac disease, it is assumed that factors other than the 15q13.3 deletion should also be involved in the appearance of the clinical traits. In this case, no additional genomic alterations were detected.

Among the additional rare rearrangements identified in fetuses with malformations, all tested were inherited from an apparently healthy progenitor, which is consistent with previous data [31], [39]. The rarity and gene content of some of these rearrangements suggest their possible pathogenic implication in congenital malformations. Nevertheless, like in some recurrent microdeletion syndromes, the existence of healthy carriers among progenitors and the adult population indicates that the rearrangements are not the only cause of the disease. Considering the epidemiologic evidence for multifactorial etiology of major malformations, these rearrangements could represent just one of the several factors involved. In this regard, a case with holoprosencephaly showed two deletions, one inherited from the mother and the other from the father, both harboring genes of the same pathway (phosphatidylinositol metabolism). Two duplication-type CNV events were also found in a fetus with hydrocephalus, although parental samples were not available to determine their inheritance pattern. However, candidate genes for brain malformation were also located in both CNVs: *DMBX1* codes for a diencephalon-mesencephalon homeobox implicated in brain development and *GPRIN2* encodes a G-protein regulated inducer of neurite overgrowth involved in formation and extension of neurite-like processes [43], [44]. Given the very low frequency of these alterations in controls, the functional relationship of altered genes and their inheritance from different progenitors at least in the first case, it is logical to propose that the double hit may have contribute to the fetal malformations by additive effect of the CNVs on altering developmental regulation. A two-hit model with several recurrent and non-recurrent CNVs has been already reported for neurobehavioral and relatively severe phenotypes [45].

In addition, we also detected the 16p13.11 1.6 Mb duplication in two cases with different phenotypes. This duplication has been found in 0.14% normal adult controls (12/8,329 controls) and in 0.27% patients with developmental delay and/or malformations (42/15,767) [34]. Given the higher frequency of this duplication in our series (2%) as well as in reported patients with developmental anomalies [41], the data highly suggest that this CNV is indeed a susceptibility variant for developmental disorders including congenital malformations. The different phenotypes related to the microduplication might also be related to the concurrence of this contiguous gene alteration with other undefined genetic or environmental second hit. Depending on the other concurrent factors that may contribute to reach the gene dysfunction threshold in a specific tissue or developmental time, the phenotype would correspond to different diseases or malformations. Although additional CNVs were not found with increased frequency in cases with respect to controls in our cohort, including the two cases with 16p13.11, secondary events of other type, such as point mutations or epimutations cannot be ruled out.

On the other hand, UPD and shared homozygosity regions were discarded by SNP array and mosaic alterations were also not identified. Although the number of samples studied is low, UPD does not seem to be a common cause of isolated congenital defects. Since DNA from the affected tissue was analyzed in 46 samples, we can also conclude that mosaicism for large rearrangements in the abnormally developed tissue is not frequent in isolated congenital malformations.

In addition to the most common aneuploidies and genomic disorders also detected by karyotyping and targeted assays, CMA significantly increases the detection yield of cryptic segmental aneuploidies in fetuses with congenital malformations. The highest yield for rare CNVs was found in samples with hypoplasia of the left/right heart, doubling the frequency of any other group of malformations and suggesting a higher genetic component for this type of malformation, which is consistent with its higher heritability [46], [47]. A higher frequency of rearrangements in patients with left heart hypoplasia comparing with controls has been recently reported, even though the difference was only statistically significant for aberrations smaller than 60 kb [48]. However, from a clinical perspective, CMA can detect the single causative alteration in a relatively low percentage of cases with isolated congenital malformations, about 2% once the most common aneuploidies and recurrent rearrangements are discarded. Therefore, although many rare CNVs detectable by CMA, like those reported here, presumably contribute to the disorder, they should be considered as variants of unknown significance until more information is available to better predict phenotype based on genotype.

Accumulation of multiple rare genomic and epigenetic variants converging to deregulate developmental genes leading to mutational loading of developmental networks may cause congenital malformations [49]. Rare copy number variants, point mutations and/or epigenetic variations, either inherited or *de novo*, can impact gene function or alter dosage and contribute to mutational load. Changes affecting multiple genes and networks related to development may induce developmental anomalies. This concept implies that if threshold levels of flux are exceeded, compensatory mechanisms may fail, leading to an inadequate development. This hypothesis has been tested in mouse model and some results suggest that the accumulation of alterations in regulatory development networks results in an inadequate development [50]. Although it is reasonable to expect homologous genes to behave similarly in humans, more evidence supporting this hypothesis is needed. Further studies, including whole genome sequencing and epigenomic analyses as well as expression profiles of genes related to development should be done in order to improve the knowledge of the etiology and the diagnostic tools for isolated congenital malformations.

## Supporting Information

Table S1 List of heart malformations present in the cohort of 33 studied fetuses with isolated congenital heart defect. RHH: right heart hypoplasia; IVC: interventricular communication; LHH: left heart hypoplasia; VSD: ventricular septal defect; D-TGA: dextro-transposition of the great arteries; L-TGA: levo-transposition of the great arteries; AVSD: atrioventricular septal defect. IAC: interatrial communication.
(DOC)

Table S2 Overview of the central nervous system malformations in 26 of the analyzed fetuses. CNS: central nervous system.
(DOC)

Table S3 Type of renal malformations observed in 9 of the studied fetuses.
(DOC)

Table S4 Overview of the affected organs and systems in fetuses with multiple malformations. CHD: congenital heart defect; CNS: central nervous system; IUGR: intrauterine growth restriction. VATER: Vertebrae, Anus, Trachea, Esophagus, and Renal. OEIS: omphalocele, exstrophy, imperforate anus, spinal defects.
(DOC)

Table S5 MLPA probes used to discard well-known genetic alterations related to MCA/MR. Hg19 assembly.
(DOC)

Table S6 MLPA probes used to validate the alterations detected by CMA and to study parental samples. Hg19 assembly.
(DOC)

Table S7 Summary of rare copy number variations >100 kb detected in samples of 168 control subjects. Hg19 assembly.
(DOC)
