# Supplementary material for: Contribution of Rare Copy Number Variants to Isolated Human Malformations
Source: PLoS One. 2012 Oct 3;7(10):e45530. doi: 10.1371/journal.pone.0045530 (PMC3463597; doi:10.1371/journal.pone.0045530)
Supplement: Table S1 — List of heart malformations present in the cohort of 33 studied fetuses with isolated congenital heart defect. RHH: right heart hypoplasia; IVC: interventricular communication; LHH: left heart hypoplasia; VSD: ventricular septal defect; D-TGA: dextro-transposition of the great arteries; L-TGA: levo-transposition of the great arteries; AVSD: atrioventricular septal defect. IAC: interatrial communication. (DOC) [file pone.0045530.s001.doc]

| **Sample** | **Tissue** | **Gender** | **Malformation** |
| --- | --- | --- | --- |
| 1 | lung | female | RHH with tricuspid valve and pulmonary artery stenosis, hypoplastic pulmonary artery and branches, membranous IVC |
| 2 | heart | female | LHH |
| 3 | heart | female | LHH |
| 4 | heart | male | LHH |
| 5 | heart | female | tricuspid valve stenosis, RHH, VSD type ostium primum, muscular VSD, mitral valve dysplasia, aneurismal dilatation of posterior left atrium |
| 6 | liver | male | LHH |
| 7 | heart | male | LHH |
| 8 | heart | male | double outlet right ventricle, D-TGA, dual left anterior descending artery, hypoplastic pulmonary artery, overriding aorta over membranous IVC |
| 9 | heart | female | L-TGA, incomplete AVSD, LHH, overriding aorta over membranous IVC |
| 10 | heart | male | double outlet right ventricle with D-TGA, extreme hypoplasia of the aortic arch, IVC, moderate hypoplasia of the supraaortic trunk |
| 11 | heart | male | double outlet right ventricle with IVC, hypoplasia of the ascending aorta |
| 12 | liver | female | L-TGA with atrioventricular discordance, subpulmonar IVC |
| 13 | liver | male | dextrocardia, D-TGA, double outlet right ventricle with IVC |
| 14 | liver | female | truncus arteriosus type II |
| 15 | heart | male | LHH |
| 16 | heart | female | tetralogy of Fallot |
| 17 | heart | male | atrioventricular discordance, right ventricle with left ventricle morphology and viceversa |
| 18 | heart | female | LHH |
| 19 | heart | male | D-TGA, muscular VSD, hypoplastic ductus arteriosus, origin of both coronary arteries from the anterior leaflet of the aortic valve, bicuspid pulmonary valve |
| 20 | liver | male | D-TGA, IAC type ostium secundum, LHH with mitral and pulmonary valves atresia, hypoplastic pulmonary artery, permeable ductus |
| 21 | heart | male | large foramen ovale, tricuspid valve atresia, VSD, TGA, pulmonary atresia, hypoplastic pulmonary artery |
| 22 | lung | female | LHH with mitral and aortic valves atresia |
| 23 | heart | female | LHH with mitral and aortic valves atresia and hypoplasia of the ascending aorta |
| 24 | heart | male | membranous IVC |
| 25 | heart | male | LHH with mitral and aortic valves hypoplasia |
| 26 | heart | female | double outlet right ventricle, large foramen ovale, VSD, coarctation of the preductal aorta |
| 27 | heart | male | hypoplasia of the ascending aorta and muscular IVC |
| 28 | heart | female | LHH with mitral and aortic valves atresia, incarceration of the left ventricle and extreme hypoplasia of the ascending aorta |
| 29 | heart | female | LHH |
| 30 | lung | female | extreme LHH with mitral and aortic valves atresia and hypoplasia of the ascending aorta |
| 31 | heart | male | LHH with mitral and aortic valves atresia, aortic valve with two leaflets and tubular hypoplasia of the aortic arch |
| 32 | heart | female | truncus arteriosus type II, absent ductus arteriosus, truncal valve with three leaflets, VSD |
| 33 | heart | male | complex cardiopathy |

*Table S1.* List of heart malformations present in the cohort of 33 studied fetuses with isolated congenital heart defect. RHH: right heart hypoplasia; IVC: interventricular communication; LHH: left heart hypoplasia; VSD: ventricular septal defect; D-TGA: dextro-transposition of the great arteries; L-TGA: levo-transposition of the great arteries; AVSD: atrioventricular septal defect. IAC: interatrial communication.
