# Supplementary material for: Contribution of Rare Copy Number Variants to Isolated Human Malformations
Source: PLoS One. 2012 Oct 3;7(10):e45530. doi: 10.1371/journal.pone.0045530 (PMC3463597; doi:10.1371/journal.pone.0045530)
Supplement: Table S2 — Overview of the central nervous system malformations in 26 of the analyzed fetuses. CNS: central nervous system. (DOC) [file pone.0045530.s002.doc]

| **Sample** | **Tissue** | **Gender** | **Malformation** |
| --- | --- | --- | --- |
| 34 | lung | female | Holoprosencephaly |
| 35 | CNS | female | Sacral neural tube defect, Arnold-Chiari malformation |
| 36 | CNS | male | Lumbosacral neural tube defect, Arnold-Chiari malformation |
| 37 | CNS | female | Lumbosacral neural tube defect, Arnold-Chiari malformation |
| 38 | liver | female | Anencephaly |
| 39 | CNS | male | Sacral myelomeningocele, Arnold-Chiari malformation |
| 40 | liver | male | Holoprosencephaly |
| 41 | liver | female | Occipital encephalocele, Arnold-Chiari malformation |
| 42 | liver | female | Biventricular hydrocephalus |
| 43 | liver | male | Lumbosacral myelomeningocele, Arnold-Chiari malformation |
| 44 | CNS | male | Bilateral ventriculomegaly |
| 45 | CNS | female | Lumbosacral myelomeningocele, Arnold-Chiari malformation |
| 46 | CNS | male | Lumbar myelomeningocele, Arnold-Chiari malformation |
| 47 | CNS | male | Biventricular hydrocephalus |
| 48 | liver | male | Lumbosacral myelomeningocele, Arnold-Chiari malformation, brachycephaly |
| 49 | liver | female | Agenesis of the corpus callosum |
| 50 | liver | female | Biventricular hydrocephalus |
| 51 | CNS | female | Lumbosacral myelomeningocele, Arnold-Chiari malformation |
| 52 | liver | male | Sacral neural tube defect, Arnold-Chiari malformation |
| 53 | heart | female | Bilateral ventriculomegaly |
| 54 | CNS | female | Lumbosacral myelomeningocele, Arnold-Chiari malformation |
| 55 | CNS | female | Lumbosacral neural tube defect, Arnold-Chiari malformation |
| 56 | CNS | female | Lumbar myelomeningocele, Arnold-Chiari malformation |
| 57 | CNS | female | Holoprosencephaly |
| 58 | CNS | female | Bilateral ventriculomegaly |
| 59  *Table S2.* Overview of the central nervous system malformations in 26 of the analyzed fetuses. CNS: central nervous system. | amniotic fluid | male | Neural tube defect |
