# Supplementary material for: Contribution of Rare Copy Number Variants to Isolated Human Malformations
Source: PLoS One. 2012 Oct 3;7(10):e45530. doi: 10.1371/journal.pone.0045530 (PMC3463597; doi:10.1371/journal.pone.0045530)
Supplement: Table S3 — Type of renal malformations observed in 9 of the studied fetuses. (DOC) [file pone.0045530.s003.doc]

| **Sample** | **Tissue** | **Gender** | **Malformation** |
| --- | --- | --- | --- |
| 60 | adrenal gland | male | Bilateral renal agenesis, bilateral agenesis of the ureters. Oligohydramnios sequence |
| 61 | lung | male | Bilateral multicystic dysplastic kidney disease with ureter dysplasia and bladder hypoplasia |
| 62 | adrenal gland | female | Bilateral renal agenesis, bilateral agenesis of the ureters. Oligohydramnios sequence |
| 63 | liver | male | Bilateral renal agenesis, bilateral agenesis of the ureters. Oligohydramnios sequence |
| 64 | lung | male | Bilateral multicystic dysplastic kidney disease with ureter dysplasia and bladder hypoplasia |
| 65 | adrenal gland | male | Congenital nephronophthisis |
| 66 | liver | male | Bilateral renal agenesis, bilateral agenesis of the ureters. Oligohydramnios sequence |
| 67 | kidney | male | Bilateral obstructive renal dysplasia |
| 68 | adrenal gland | male | Bilateral renal agenesis, bilateral agenesis of the ureters. Oligohydramnios sequence |

*Table S3.* Type of renal malformations observed in 9 of the studied fetuses.
