# Supplementary material for: Contribution of Rare Copy Number Variants to Isolated Human Malformations
Source: PLoS One. 2012 Oct 3;7(10):e45530. doi: 10.1371/journal.pone.0045530 (PMC3463597; doi:10.1371/journal.pone.0045530)
Supplement: Table S4 — Overview of the affected organs and systems in fetuses with multiple malformations. CHD: congenital heart defect; CNS: central nervous system; IUGR: intrauterine growth restriction. VATER: Vertebrae, Anus, Trachea, Esophagus, and Renal. OEIS: omphalocele, exstrophy, imperforate anus, spinal defects. (DOC) [file pone.0045530.s004.doc]

| **Sample** | **Tissue** | **Gender** | **CHD** | **CNS** | **IUGR** | **Craniofacial** | **Skeletal** | **Urogenital** | **Others** |
| --- | --- | --- | --- | --- | --- | --- | --- | --- | --- |
| **69** | heart | male | X |  |  |  |  |  | Esophageal atresia, anal imperforation (VATER) |
| **70** | heart | female | X |  |  |  |  |  | Asplenia |
| **71** | liver | female |  | X |  |  | X | X | Craniosynostosis |
| **72** | liver | male |  |  |  | X | X |  | Lymphangioma |
| **73** | liver | female | X |  |  |  |  | X |  |
| **74** | spleen | female | X | X |  |  |  |  | Omphalocele |
| **75** | liver | male | X |  |  |  |  | X |  |
| **76** | liver | female |  | X |  | X | X |  |  |
| **77** | liver | male | X | X | X | X | X | X |  |
| **78** | liver | male | X |  |  |  |  | X |  |
| **79** | liver | male | X |  |  |  |  |  | Asplenia |
| **80** | liver | female | X | X |  | X |  |  |  |
| **81** | liver | male |  |  | X | X |  | X |  |
| **82** | lung | male |  |  |  | X | X | X |  |
| **83** | liver | female |  | X |  | X |  |  | Hairy polyp, bilateral pieloureteral duplication |
| **84** | lung | male | X |  |  |  |  | X |  |
| **85** | liver | female | X |  |  |  |  |  | Accessory spleen, pulmonary hypoplasia |
| **86** | liver | female |  | X |  |  |  | X | Accessory spleen, malformation of the adrenal gland |
| **87** | liver | female |  | X |  |  |  | X |  |
| **88** | heart | male | X |  |  |  | X |  |  |
| **89** | liver | female |  |  |  |  |  |  |  |
| **90** | liver | male | X |  |  | X |  |  | Two accessory spleen, bilateral adrenal neuroblastoma |
| **91** | liver | female |  |  |  |  | X | X | OEIS complex |
| **92** | adrenal gland | male |  |  |  |  |  | X | Anal imperforation, rectovesical fistula |
| **93** | liver | female | X | X | X | X | X | X |  |
| **94** | liver | female | X |  |  | X |  | X | Diaphragmatic hernia |
| **95** | liver | female | X |  |  |  |  | X | Omphalocele, anal imperforation |

*Table S4.* Overview of the affected organs and systems in fetuses with multiple malformations. CHD: congenital heart defect; CNS: central nervous system; IUGR: intrauterine growth restriction. VATER: Vertebrae, Anus, Trachea, Esophagus, and Renal. OEIS: omphalocele, exstrophy, imperforate anus, spinal defects.
