# Supplementary material for: Contribution of Rare Copy Number Variants to Isolated Human Malformations
Source: PLoS One. 2012 Oct 3;7(10):e45530. doi: 10.1371/journal.pone.0045530 (PMC3463597; doi:10.1371/journal.pone.0045530)
Supplement: Table S5 — MLPA probes used to discard well-known genetic alterations related to MCA/MR. Hg19 assembly. (DOC) [file pone.0045530.s005.doc]

| **Gene**  Table 4. Summary of copy number variations detected in 33 samples of fetuses with isolated CHD. Hg18 assembly. | **Chr** | **Start** | **End** | **Syndrome related** | **Hybridization sequence** |
| --- | --- | --- | --- | --- | --- |
| *WBSCR1* | 7 | 73604582 | 73604629 | Williams-Beuren sd | GGTAGCTCTCGAGAATCTAGAGGTGGATGGGATTCCCGGGATGACTTC |
| *HIRA* | 22 | 19318971 | 19319021 | DiGeorge sd | CCCTCAGGATGTCGAGCTGTTCCTGACACTCGGTGAAGAGGCGCTGGAATC |
| *NSD1* | 5 | 176700712 | 176700762 | Sotos sd | CCCAAAAAGAGCTAAGACAGCTGCAGGAAGACCGAAAGAATGACAAGAAGC |
| *SNRNP* | 15 | 25200680 | 25200734 | Prader-Willi sd | AGGGGGTGTTGAGCGCAGGTAGGTGTATAATAGTGACCACTGCGTGGTGGAGCAG |
| *ARIH1* | 15 | 72855773 | 72855832 | 15q24 del sd | CCACCATGTTGTTAAAGTCCAATATCCTGATGCTAAACCTGTTCGCTGCAAATGTGGGCG |
| *TRIP3* | 17 | 34851076 | 34851139 | Mental retardation | CAACATTAAGAAGCTTATTGCTCAATCCACACCTCAGGCAGTTGATGGTCAACCTCGATCAGGG |
| *PML* | 15 | 74290611 | 74290671 | 15q24 del sd | CAAAGAGTCGGCCGACTTCTGGTGCTTTGAGTGCGAGCAGCTCCTCTGCGCCAAGTGCTTC |
| *COPS3* | 17 | 17179360 | 17179431 | Smith-Magenis sd | CTTGCGAAGAACTTATCCCATCTGGACACTGTGCTCGGGGCTCTGGATGTACAAGAACACTCCTTGGGCGTC |
| *BAZ1B* | 7 | 72925125 | 72925198 | Williams-Beuren sd | AGTACTCTTGCACGTCCAAATGCGCTCACTGTACCTTTCCAAGCGGGCTTCATACTCTCTGTTGGCAGTAGTTC |
| *MAPT* | 17 | 44102520 | 44102589 | 17q21.31 del sd | CTGGGACTTTAGGGCTAACCAGTTCTCTTTGTAAGGACTTGTGCCTCTTGGGAGACGTCCACCCGTTTC |
| *BCL9* | 1 | 147083562 | 147083622 | 1q21.1 sd | CTGTTTCTGCTGCAACCCGAGAGGAACTCGGTGAGCCTGTCCCGTTTGTGACTGCAAGCTC |
| *CDH9* | 5 | 26906872 | 26906944 | Control probe | CATCTGTTATACAAGTAACTGCAACAGATGCAGATGACGCCAACTATGGAAATAGTGCCAAAGTGGTCTATAG |
| *KIAA0427* | 18 | 46383961 | 46384012 | Control probe | CTTGCAATCTCAGGATGTGAAGGAAGATGCTGTCCTTTGCTGCTCTATGGAG |
| *GNB1L* | 22 | 19789603 | 19789662 | DiGeorge sd | GAAGGTGTGCAGCCGCATCGCCTGCCATGAGGAGCCCGTCATGGACCTTGACTTTGACTC |
| *FLJ20436* | 12 | 49065580 | 49065646 | Control probe | GCCTAGAGCTTCATGTTCCACTTTGCGATTATGTAAGTATCGGCGCTTCTTCTCCTTGAGCAGATGC |
| *SKI* | 1 | 2161109 | 2161169 | 1p36 syndrome | CGCTGCCTGGACGACGTGAAGGAGAAATTCGACTATGGCAACAAGTACAAGCGGCGGGTGC |
| *TP73* | 1 | 3624031 | 3624090 | 1p36 syndrome | CCTGTAACAGGACACCTCCTAGACGGGACAGGACGACTGACTGTGTGTGTTTCCCCCTCC |

*Table S5.* MLPA probes used to discard well-known genetic alterations related to MCA / MR. Hg19 assembly.
