# Supplementary material for: Contribution of Rare Copy Number Variants to Isolated Human Malformations
Source: PLoS One. 2012 Oct 3;7(10):e45530. doi: 10.1371/journal.pone.0045530 (PMC3463597; doi:10.1371/journal.pone.0045530)
Supplement: Table S6 — MLPA probes used to validate the alterations detected by CMA and to study parental samples. Hg19 assembly. (DOC) [file pone.0045530.s006.doc]

| **Gene** | **Chr** | **Start** | **End** | **Hybridization sequence** |
| --- | --- | --- | --- | --- |
| ***KLF13*** | 15 | 31666123 | 31666182 | GGTATCTTGCCAGAGATACCTGTTTTGATGAGTACCTATTTTGATGCAAAGGAACGGCCC |
| ***FOXC2*** | 16 | 86602268 | 86602335 | CAGCAGCAAACTTTCCCCAACGTGCGGGAGATGTTCAACTCCCACCGGCTGGGGATTGAGAACTCGAC |
| ***OSGIN*** | 16 | 83982733 | 83982798 | GAAAGTCCACGTCTCGCCCCACAGTTCCCCATATCAGCAAATCTTCAAATTCTAGAACAAGGTCAG |
| ***DIAPH3*** | 13 | 60584710 | 60584762 | CTTATTGGCCAAAGCCGTGGATCCCAGACACCCCAATATGATGACAGATGTGG |
| ***USP46*** | 4 | 53468131 | 53468190 | GGCCCTGCACCTAAAGCGGTTCAAGTACATGGAGCAGCTGCACAGATACACCAAGCTGTC |
| ***GPLD1*** | 6 | 24447129 | 24447190 | GCAGCCAACTGGACGGTGAGAGGCGAGGAAGACTTCTCCTGGTTTGGATATTCCCTTCACGG |
| ***PIK3C2G*** | 12 | 18499652 | 18499715 | GCAGATTTTCAGCCTGTAAATGTACCTAGATGCACTTCCTATCTAAATCCCGGGCTTCCTTCCC |
| ***CDH19*** | 18 | 64212057 | 64212119 | CTGAATCTGCACCCACTGGGACTTCTATAGGAACAATCATGGCATATGATAATGACATAGGAG |
| ***CNTN4*** | 3 | 3081837 | 3081894 | CGTGTTCAGGAATGAGAGCGTGCACCCCTTCTCTCCCTTTGAGGTTAAAGTAGGTGTC |

*Table S6.* MLPA probes used to validate the alterations detected by CMA and to study parental samples. Hg19 assembly.
