# Supplementary material for: Contribution of Rare Copy Number Variants to Isolated Human Malformations
Source: PLoS One. 2012 Oct 3;7(10):e45530. doi: 10.1371/journal.pone.0045530 (PMC3463597; doi:10.1371/journal.pone.0045530)
Supplement: Table S7 — Summary of rare copy number variations >100 kb detected in samples of 168 control subjects. Hg19 assembly. (DOC) [file pone.0045530.s007.doc]

| **Sample** | **Gain/Loss** | **Region** | **Length (kb)** | **Start** | **End** | **Genes in the region** | **Control frequency (10,320)** |
| --- | --- | --- | --- | --- | --- | --- | --- |
| 1101 | Gain | 2p16.1 | 115.779 | 56606229 | 56722007 | *CCDC85A* | 0 |
| 1007 | Gain | 5q35.1 | 289.45 | 169441749 | 169731198 | *DOCK2,FOXI1,LCP2,LOC133874* | 0 |
| 101 | Loss | 6q22.33 | 171.412 | 127983009 | 128154420 | *THEMIS* | 0 |
| 1091 | Gain | 10p15.1 | 122.207 | 5016797 | 5139003 | *AKR1C1, AKR1C2, AKR1C3, LOC100134257* | 0 |
| 1087 | Loss | 4q12 | 312.257 | 53906416 | 54218672 | *SCFD2* | 0 |
|  | Gain | 3p25.3 | 164.621 | 9900524 | 10065144 | *CIDEC, CIDECP, CRELD1, IL17RC, IL17RE, JAGN1, LOC401052, PRRT3, TMEM111* | 0 |
| 119 | Gain | 12p11.21 | 194.756 | 32871481 | 33066236 | *DNM1L, PKP2, YARS2* | 0 |
| 1024 | Loss | 9p22.1 | 680.069 | 18208852 | 18888920 | *ADAMTSL1* | 0 |
| 1030 | Gain | 10q23.33 | 134.131 | 96626682 | 96760812 | *CYP2C9* | 0 |
| 1032 | Gain | 8p23.1 | 428.039 | 8215349 | 8643387 | *CLDN23, MFHAS1, PRAGMIN* | 0 |
| 14 | Gain | 4p15.32 | 736.736 | 16842680 | 17579415 | *CLRN2, LAP3, LDB2, QDPR* | 1 |
| 1041 | Gain | 16p12.3 | 268.802 | 18792147 | 19060948 | *ARL6IP1, RPS15A, SMG1, TMC7* | 1 |
| 1010 | Gain | 22q11.23 | 538.044 | 23111199 | 23649242 | *BCR, GNAZ, RAB36, RTDR1* | 1 |
| 136 | Gain | 18q12.2 | 674.203 | 34279819 | 34954021 | *BRUNOL4, C18orf10, FHOD3, KIAA1328* | 1 |
| 1064 | Gain | 6q15 | 326.983 | 90590332 | 90917314 | *BACH2, GJA10* | 1 |
| 1006 | Gain | 13q12.11 | 144.699 | 20066736 | 20211434 | *MPHOSPH8,TPTE2* | 1 |
| 1082 | Gain | 4q31.3 | 402.135 | 151668146 | 152070280 | *LRBA, RPS3A, SH3D19, SNORD73A* | 1 |
| 1115 | Gain | 3q13.12 | 106.25 | 107441005 | 107547254 | *BBX* | 1 |
| 113 | Gain | 5q35.2 | 317.302 | 175467619 | 175784920 | *C5orf25, FAM153B, KIAA1191* | 1 |
| 1029 | Gain | 12q24.33 | 454.025 | 132527235 | 133037139 | *DDX51, EP400, EP400NL, GALNT9, LOC100130238, NOC4L* | 1 |
| 1014 | Gain | 5q11.2 | 504.677 | 55415101 | 55919777 | *ARL15* | 1 |
| 1015 | Loss | 13q12.13 | 200.368 | 26174679 | 26375046 | *ATP8A2* | 1 |
| 1106 | Gain | 2p21 | 564.149 | 45409988 | 45974136 | *PRKCE,SRBD1* | 2 |
| 1020 | Gain | 10q26.3 | 192.786 | 134568387 | 134761172 | *C10orf92, C10orf93, INPP5A, NKX6-2* | 2 |
| 152 | Gain | 5q22.3 | 437.6 | 115071808 | 115509419 | *AP3S1, ATG12, CDO1, COMMD10, LVRN* | 2 |
| 1099 | Loss | 11q21 | 110.245 | 95933123 | 96043367 | *MAML2* | 4 |
| 1045 | Gain | 7p15.1 | 327.356 | 29209352 | 29536707 | *CHN2* | 4 |
| 1061 | Gain | 8q21.3 | 156.332 | 87177637 | 87333968 | *SLC7A13* | 4 |
| 1080 | Gain | 1p22.1 | 351.535 | 92273644 | 92625178 | *BRDT, BTBD8, EPHX4, TGFBR3* | 4 |
| 1034 | Gain | 16q23.1 | 458.538 | 76453043 | 76911580 | *CNTNAP4* | 5 |
| 1049 | Loss | 17q24.3 | 141.176 | 67169500 | 67310675 | *ABCA10, ABCA5* | 5 |
|  | Loss | 15q25.3 | 254.344 | 87870024 | 88124367 | *NCRNA00052* | 0 |

*Table S7*. Summary of rare copy number variations >100kb detected in samples of 168 control subjects. Hg19 assembly.
